# Plant virus metagenomics: what we know and why we need to know more

**DOI:** 10.3389/fpls.2014.00150

**Published:** 2014-04-16

**Authors:** Anthony H. Stobbe, Marilyn J. Roossinck

**Affiliations:** ^1^Department of Plant Pathology and Environmental Microbiology, Center for Infectious Disease Dynamics, Pennsylvania State UniversityUniversity Park, PA, USA; ^2^State Agricultural Biotechnology Centre, Murdoch UniversityPerth, WA, Australia

**Keywords:** ecogenomics, virus ecology, wild plant viruses, virus biodiversity, bioinformatics

## Introduction

In the past decade the concept of plant viruses as strictly disease-causing entities has been challenged. While the most well-studied and obvious interactions between plants and viruses are related to disease, there are several examples of mutualistic relationships between plants and viruses, both indirect and direct. These mutualistic interactions have not been fully explored, and many questions remain unanswered. One problem is the lack of knowledge of plant viruses in nature. Metagenomic surveys have estimated that only a small fraction of virus species are known. Additionally, globalization has led to the increased movement of plant material and virus movement. As viruses move from one area to another, new potential hosts offer the possibility of new interactions, both negative and positive.

## Beneficial plant-virus interactions

Viruses have been associated with plant disease since they were first described in 1898 (Beijerinck, 1898), but in recent years viruses with positive impacts on the plant hosts they are associated with also have been described. Negative interactions are mostly studied as disease symptoms such as stunting or necrosis, and the vast majority of virus research has focused on the disease aspect of these interactions. Beneficial interactions involve environmental protection to the host plant, protection against other pathogens, or control of plant responses to nutritional needs (reviewed in Roossinck, 2011). Plant viruses confer drought and cold tolerance to plants as conditional mutualists: the plant is harmed by the viruses under normal conditions, but benefited under extreme conditions. This was demonstrated for several different viruses and plant hosts (Xu et al., 2008). Mild strains of plant viruses protect plants from more severe isolates, a phenomenon known as cross-protection (Fraser, 1998) that led to the initial generation of virus-induced pathogen protection in transgenic plants. Endogenous pararetroviral elements in plants can confer resistance to exogenous viruses (Staginnus et al., 2007). The coat protein gene of a persistent virus in white clover affects the development of nodules under varying nitrogen levels, and this could be transferred to other legumes (Nakatsukasa-Akune et al., 2005). *Curvularia* thermal tolerance virus is a mycovirus that infects a plant fungal endophyte, *Curvularia protuberata*. When both virus and fungus are present in hot springs panic grass (*Dichanthelium lanuginosum*) the holobiont is able to grow in soil temperatures up to 65°C (Márquez et al., 2007). Many more examples of mutualistic viruses can be found in other hosts (Roossinck, 2011). In addition, viruses are important in population control of their hosts, and marine viruses are probably extremely important to the movement of carbon and trace elements in the microbiome of the oceans (Danovaro et al., 2011).

## Plant virus ecology and evolution

The existence of plant-virus mutualistic relationships should not be surprising when one considers the numerous examples of mutualistic relationships between plants or animals and other microbes. Despite examples, there has been very little focus on exploring mutualistic relationships among plants and viruses. Viruses are also involved in the complex interactions between plants and insects, and can alter insect feeding behavior, fecundity, and ability to invade new territory (reviewed in Roossinck, 2013).

Further complicating our understanding of plant-virus interactions is the role globalization has on the relationships between viruses and their plant hosts. Viruses are not stationary, and their movement geographically and between host species can have drastic effects on the ecology of a given area. Climate change can alter the behavior of many virus vectors, promoting the spread of viral distribution across a larger geographic area (Lebouvier et al., 2011). A prime example of the impact of viruses on plant species balance is the well-studied beneficial effect the luteoviruses *Barley yellow dwarf virus* and *Cereal yellow dwarf virus* had on the invasive annual grasses in California grasslands (Malmstrom et al., 2005).

Using the estimation that over 20,000 microbes have invaded the United States (Pimentel et al., 2005) as a measure of virus movement worldwide, it is reasonable to suggest that this significant movement of viruses gives the opportunity of a virus jumping from one plant host species to another, which in turn leads to new plant-virus interactions. Metagenomic surveys can be useful for nations who are interested in protecting their crops against invasive diseases (MacDiarmid et al., 2013). A working knowledge of the geographic location, host range, and potential effects of plant viruses can assist such nations in developing effective policies that distinguish those viruses that will have negative economic impacts from viruses which are benign or even beneficial.

Viruses impact the evolution of plants at many levels, and plants clearly affect the populations of viruses that infect them. There are examples of specific interactions between plants and viruses, such as the silencing suppression genes found in many RNA viruses. While not as prevalent as in animals or bacteria, there have been instances of horizontal gene exchange from viruses to plants. Repeat sequences of geminiviruses have been found in *Nicotiana spp*. (Bejarano et al., 1996; Ashby et al., 1997), and pararetroviruses are frequently found integrated into plants (Hohn et al., 2008). Sequences from cytoplasmic RNA viruses are found in plant genomes (Liu et al., 2010; Chiba et al., 2011). There is also evidence that Closterovirus genes have integrated into the mitochondria of grapevines, *Vitis vinifera* (Goremykin et al., 2009). Viruses have been said to be responsible for a large amount of genetic flow in several different systems (Bock, 2010; Liu et al., 2010; Wu and Zhang, 2011). This in turn would increase the genetic plasticity of the hosts offering the opportunity for novel interactions to take place.

## We don't know what is out there

In the past decade there have been a few metagenomic type surveys exploring plant virus biodiversity in wild plants, insects, and a few other environments (Wren et al., 2006; Roossinck et al., 2010; Ng et al., 2011; Roossinck, 2012). Some of these studies have used a more ecological approach, “ecogenomics,” that looks at the viral populations in individuals rather than in the entire environment that is typical of metagnomic studies (Roossinck et al., 2010). The most surprising result is that we know very little of the size and diversity of plant virus families. These surveys have revealed that the true diversity of virus species is much larger than earlier estimates, with the discovery of new virus isolates, species, families, and even higher level virus groups (Labonté and Suttle, 2013). An additional surprising result is that viruses in wild plants do not cause any visible symptoms. With the knowledge of how little we know of the biodiversity of viruses, new techniques, methods, and questions need to be developed in order to detect and identify these new viruses. In addition, the full extent of plant-virus interactions cannot be fully studied until we have a better understanding of the ecology of plant viruses. While the metagenomic surveys are a start, there are still many challenges ahead.

The viruses found using metagenomic sequencing data can be described in three different ways: (1) Known-knowns: virus species or isolates that are already known to be in the environment being surveyed; (2) Unknown-knowns: new virus species or isolates of a known family, or known viruses that have not been found previously in the surveyed environment and; (3) Unknown-unknowns: viruses that are completely novel and share little to no sequence similarity with other known viruses. Sequencing data for each instance can be analyzed differently based on the questions being addressed. The removal of non-viral sequences from the sample either before or after sequencing will, of course, increase the chances of identifying viruses within a metagenomic sequence dataset, so care should be taken in both sample preparation before sequencing and manipulation of sequence data after sequencing. Methods to enrich for plant viral-specific sequences include isolation of virus-like particles (Muthukumar et al., 2009), enrichment for double-stranded RNA (Roossinck et al., 2010), and the use of siRNAs (Kreuze et al., 2009). All of these methods have strengths and weaknesses, but the use of double-stranded RNA has given the deepest analyses so far. For known-knowns and unknown-knowns, screening of the sequence dataset for the presence of known viruses can drastically reduce the amount of time needed for analysis and as such detection and identification of viruses (Stobbe et al., 2013).

The large amount of sequencing data that shares little or no nucleotide similarity with known sequences in curated databases such as GenBank suggests that there are still many unknown microbes that have yet to be described, with unknown-unknown viruses likely to be prevalent among them. These unknown sequences continue to be difficult to identify and will require new and novel methods to assign to a taxon. There have been significant efforts to describe these unknown-unknowns in different environments, with new analysis methods tailored for virus discovery either by sequence similarity or by clustering genes (Williamson et al., 2008; Kristensen et al., 2010; Wu et al., 2010; Ames et al., 2013; Labonté and Suttle, 2013). Protein sequences expressed by viral-specific genes, such as the RNA dependent RNA polymerase (RdRp), can be used to detect both unknown-knowns and unknown-unknowns (Kristensen et al., 2010).

Unknown-unknown viruses share little or no sequence similarity with known viruses, so quality *de novo* assembly is essential as genome mapping or BLAST assisted assembly is not an option. Assembly programs tailored for virus *de novo* assembly have been created, and modifications to assembly processes have been used to generate fully assembled genomes of extremely low titer microbes (Albertsen et al., 2013). Additionally, new exciting sequencing platforms, such as the Oxford Nanopore, offer the ability to sequence an entire viral genome in a single read (Schneider and Dekker, 2012). This ability removes the need for assembly altogether. However, even with a perfectly assembled genome, the need to identify the genome is still there. By clustering the unknown sequences, the biodiversity of a given environment can be estimated, and this has led to the discovery of new families of single stranded DNA viruses (Labonté and Suttle, 2013).

## Conclusions

There is still much to be discovered on the topic of plant-microbe interactions, and of plant-virus interactions in particular. Metagenomics offers us a unique tool to elucidate the current state of viruses in plants and the role viruses play in these interactions. When these studies are done on individual plant samples rather than pooled samples from a larger environment, a system known as “ecogenomics” (Roossinck et al., 2010), they provide meaningful data for deeper ecological analyses of the distribution of viruses and potential host- or environmental-specific interactions.

### Conflict of interest statement

The authors declare that the research was conducted in the absence of any commercial or financial relationships that could be construed as a potential conflict of interest.
